# Generation and Differentiation of Adult Tissue-Derived Human Thyroid Organoids

**DOI:** 10.1016/j.stemcr.2021.02.011

**Published:** 2021-03-11

**Authors:** Vivian M.L. Ogundipe, Andries H. Groen, Nynke Hosper, Peter W.K. Nagle, Julia Hess, Hette Faber, Anne L. Jellema, Mirjam Baanstra, Thera P. Links, Kristian Unger, John T.M. Plukker, Rob P. Coppes

**Affiliations:** 1 Department of Biomedical Sciences of Cells and Systems, Section of Molecular Cell Biology, University of Groningen, University Medical Center Groningen, Groningen 9713 GZ, the Netherlands; 2 Department of Surgical Oncology, University of Groningen, University Medical Center Groningen, Groningen 9713 GZ, the Netherlands; 3 Department of Radiation Oncology, University of Groningen, University Medical Center Groningen, Groningen 9713 GZ, the Netherlands; 4 Department of Endocrinology, University of Groningen, University Medical Center Groningen, Groningen 9713 GZ, the Netherlands; 5 Research Unit Radiation Cytogenetics, Helmholtz Zentrum München, German Research Center for Environmental Health GmbH, Neuherberg 85764, Germany; 6 Department of Radiation Oncology, University Hospital, LMU Munich, Munich 81377, Germany

**Keywords:** hypothyroidism, thyroid-tissue-derived organoids, stem cells, regeneration, thyroid cancer, xeno-transplanation, thyroglobulin

## Abstract

Total thyroidectomy as part of thyroid cancer treatment results in hypothyroidism requiring lifelong daily thyroid hormone replacement. Unbalanced hormone levels result in persistent complaints such as fatigue, constipation, and weight increase. Therefore, we aimed to investigate a patient-derived thyroid organoid model with the potential to regenerate the thyroid gland. Murine and human thyroid-derived cells were cultured as organoids capable of self-renewal and which expressed proliferation and putative stem cell and thyroid characteristics, without a change in the expression of thyroid tumor-related genes. These organoids formed thyroid-tissue-resembling structures in culture. (Xeno-)transplantation of 600,000 dispersed organoid cells underneath the kidney capsule of a hypothyroid mouse model resulted in the generation of hormone-producing thyroid-resembling follicles. This study provides evidence that thyroid-lineage-specific cells can form organoids that are able to self-renew and differentiate into functional thyroid tissue. Subsequent (xeno-)transplantation of these thyroid organoids demonstrates a proof of principle for functional miniature gland formation.

## Highlights

• Thyroid-organoid-derived cells can self-renew and form all tissue lineages
• Thyroid organoids express putative stem cell markers and no tumor gene expression
• Maturation of these organoids leads to formation of tissue-resembling mini-glands
• (Xeno-)transplantation into hypothyroid mice regenerates thyroid-resembling tissue

## Graphical abstract

**Figure:**
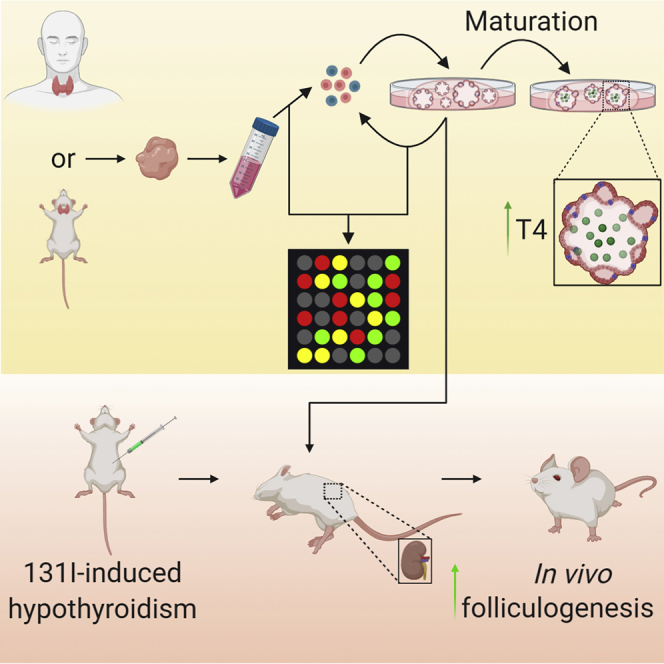

## Teaser

In this article, Ogundipe and colleagues demonstrate that thyroid gland-derived cells can be cultured as organoids for a prolonged time and differentiate *in vitro* to form hormone-producing mini-glands. (Xeno-)transplantation of dissociated organoids into hypothyroid mice allows generation of hormone-producing tissue-resembling follicles. These findings indicate that thyroid-lineage-specific cells contain proliferative potential to form thyroid-derived organoids capable of generating functional tissue.

## Introduction

Hypothyroidism results from the lack of thyroid hormones due to thyroid surgery, external beam irradiation, agenesis, or thyroid autoimmunity. About 4.6% of the US population ages 12 and older have hypothyroidism (Garber et al., 2013). Globally, hypothyroidism is among the most common diseases in the general population and increases the risk of cardiovascular, metabolic, depressive, and anxiety disorders (Wiersinga, 2014). Furthermore, hypothyroidism requires lifelong daily thyroid hormone replacement therapy consisting of levothyroxine (LT4).

Thyroid hormones are essential for the development of several tissues such as the brain, skeletal muscles, and bones. Moreover, they are required for lipid metabolism, proper regulation of tissue maintenance, and thermogenesis (Visser, 2018). In healthy tissue, free T4 is secreted from the thyroid gland and converted into the bioactive triiodothyronine (T3) in thyroid peripheral tissues. This accounts for 80% of T3 secretion, while the remaining 20% is secreted directly from the thyroid gland itself. Almost 10% of the adult patients suffer from persistent severe complaints, which are largely related to thyroid hormone replacement therapy and have a major impact on quality of life (Husson et al., 2013). In addition to patients' non-compliance, poor LT4 uptake may be caused by gastrointestinal disorders or drug interactions (Benvenga, 2013). Therefore, hormonal replacement therapy is an imperfect solution to a failing or dysfunctional organ system, resulting in imbalances in the hormonal equilibrium. Insufficient levels of thyroid hormone lead to fatigue, feeling cold, constipation, and weight gain, whereas high levels could lead to cardiovascular diseases (Elnakish et al., 2015; Klein Hesselink et al., 2013) or increased osteoporosis (Gorka et al., 2013). This is even more critical in children, who need optimal thyroid hormone levels to support neurological development and growth. Therefore, a form of regenerative medicine to restore normal thyroid function might be an attractive alternative to drug treatment.

There is mounting evidence for the existence of adult mouse (Hoshi et al., 2007) and human (Gianì et al., 2015; Lan et al., 2007; Thomas et al., 2006) thyroid stem cells. However, a specific thyroid stem cell marker has yet to be discovered (Nilsson and Fagman, 2017). Dispersed human thyrocytes have been shown to reconstitute human thyroid follicles *in vivo* (Martin et al., 1993), while *in vitro* the thyroid follicles are capable of thyroid hormone secretion (Kraiem et al., 2000). Mouse (Antonica et al., 2012; Arufe et al., 2006, 2009; Jiang et al., 2010; Lin et al., 2003; Ma et al., 2013; Risheng et al., 2009) embryonic stem cells (ESCs) and human (Ma et al., 2015) ESCs have the capacity to differentiate toward an endocrine lineage into T4-producing thyrocyte-like cells *in vitro* and *in vivo*. Furthermore, these mouse ESCs (Souza Do Rósario et al., 2005), as well as mouse and human induced pluripotent stem cell (iPSC)-derived thyroid progenitors (Kurmann et al., 2015; Serra et al., 2017), could be directed toward differentiation to yield fully mature thyroid-hormone-producing thyroid follicular organoids. However, ESC- and iPSC-derived regeneration is hampered by ethical and practical difficulties. Autologous adult tissue-derived stem cells could circumvent these issues.

Tissue-resembling organoids and mini-organs have been cultured from many tissues, such as the liver (Huch et al., 2013), intestine (Sato et al., 2009), salivary gland (Maimets et al., 2016), and endometrium (Turco et al., 2017). These organoids contain tissue-specific stem cells and differentiated cells, and resemble the organ from which they are derived. Moreover, 3D organoid culture systems allow the expansion of genetically and phenotypically organ-specific stable adult stem cells, thus eliminating the risk of *in vitro* transformation (Sato et al., 2011). Interestingly, exocrine salivary gland organoid/spheroid-derived cells are able to rescue salivary gland from irradiation damage upon (xeno-)transplantation (Maimets et al., 2016; Pringle et al., 2016). To date, thyroid gland-derived organoids containing adult stem cells with the potential to produce functional follicles have not been developed.

In this study, we isolated and characterized cells from mouse and human thyroid gland tissue and developed an *in vitro* 3D culture system to allow the culture of thyroid gland-resembling organoids with a subpopulation of cells possessing potential stem cell characteristics. The ability of these organoids to develop into thyroid gland tissue underneath the kidney capsule was shown using a hypothyroid mouse model.

## Results

### Characterization and Self-Renewal Capacity of Thyroid Gland Cells

**Figure 1:**
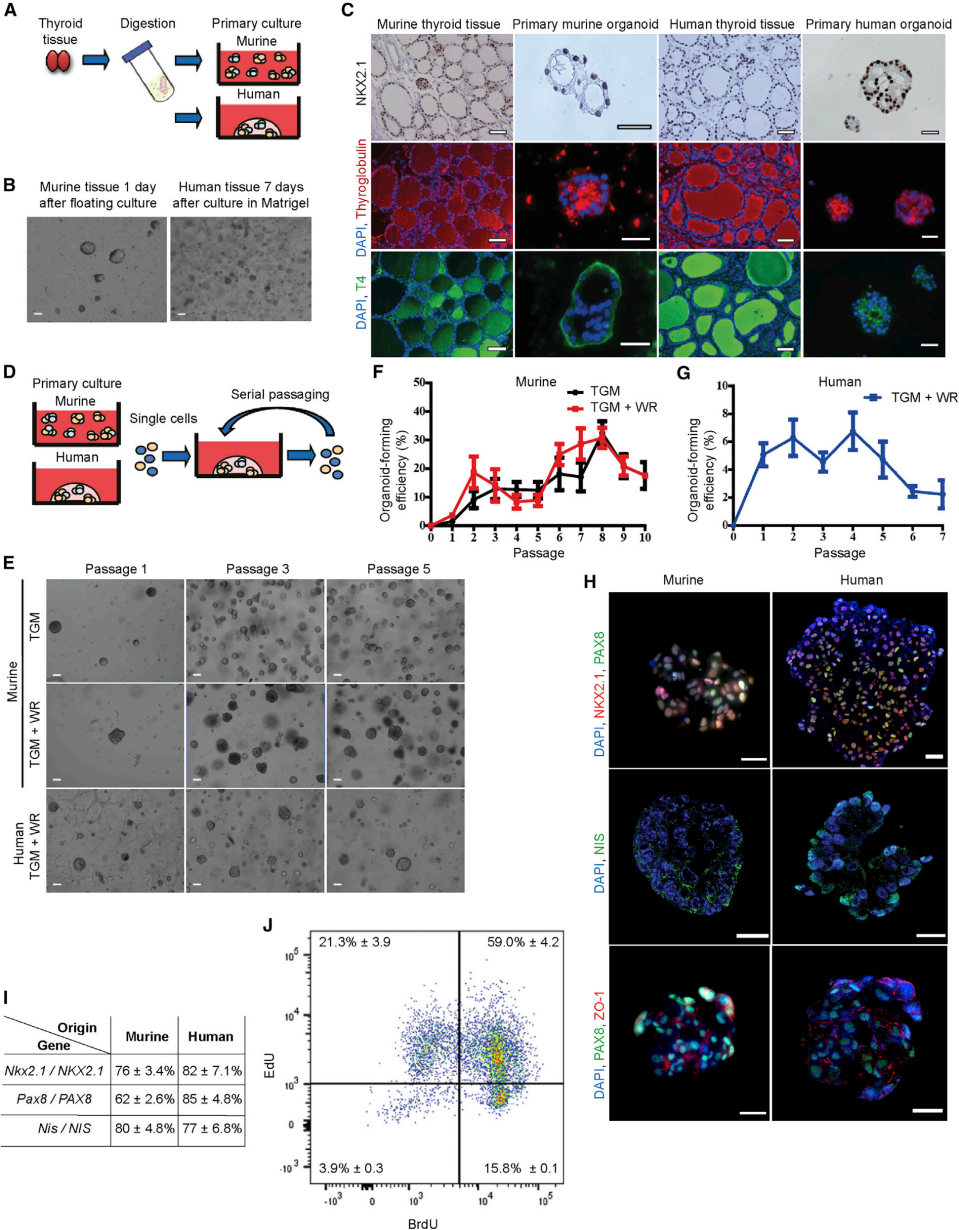
Establishment and Characterization of Murine and Human Thyroid Gland Organoid Cultures (A) Schematic representation of murine and human thyroid primary cell culture. Thyroid gland tissue was mechanically and enzymatically digested and resuspended in culture medium or seeded in Matrigel. (B) Primary murine thyroid spheres after 1 day in floating culture, and primary human thyroid organoids after 7 days in culture in Matrigel. Scale bars, 100 μm. (C) NKX2.1, thyroglobulin, and T4 staining of murine and human tissue and primary murine spheres after 1 day in floating culture and primary human thyroid organoids after 7 days in culture in Matrigel shows a nuclear staining for NKX2.1 and staining for thyroglobulin and T4. Scale bars, 50 μm for tissue and 25 μm for organoids. (D) Schematic representation of the self-renewal assay. Primary spheres (murine and human) were digested into single cells and replated in Matrigel. Organoids were passaged every 7 (murine) or 14 days (human). (E) Murine thyroid gland organoids at passages 1, 3, and 5 cultured in thyroid gland medium (TGM) or cultured in TGM supplemented with Wnt and R-spondin1 (TGM + WR) and human thyroid gland organoids at passages 1, 3, and 5. Scale bars, 100 μm. (F) Organoid-forming efficiency of murine thyroid gland cells in TGM (n = 15 independent self-renewal assays) and in TGM + WR (n = 6 independent self-renewal assays) up to passage 10. (G) Organoid-forming efficiency of human thyroid gland cells during multiple passages (n = 17 independent donor biopsies used for the self-renewal assay). (H) Representative confocal images of immunofluorescence staining for NKX2.1, PAX8, NIS, and ZO-1 in murine and human organoids. All organoids were from passage 2; scale bars, 20 μm. (I) Quantification indicating percentage of cells expressing thyroid-specific genes *NKX2.1*, *PAX8*, and *NIS* in murine and human organoids from passage 2. (J) Dual-pulse labeling using EdU and BrdU in passage 3 murine thyroid organoids to identify DNA template strand segregation during cell division. All data are represented as mean ± SEM. See also Figure S1 and Video S1.

To initiate murine thyroid tissue culture, we mechanically and enzymatically digested the glands from three mice, resulting in dispersed cells that formed spheroids when resuspended in defined thyroid gland medium (TGM; Figures 1A and 1B). Similarly, we mechanically and enzymatically digested healthy human thyroid gland tissue, but the dispersed cells were seeded directly into Matrigel. Upon polymerization of the extracellular matrix, a defined human TGM with the addition of Wnt and R-spondin1 (TGM + WR) was added, resulting in sphere formation within 7 days (Figures 1A and 1B). Wnt and R-spondin1 are known to play a crucial role in self-renewal of multiple types of adult stem/progenitor cells (de Lau et al., 2012). Next, we assessed expression of the thyroid-specific genes *NKX2*.1 (also known as thyroid transcription factor-1), *PAX8*, *THYROGLOBULIN* (*TG*), thyroid-stimulating hormone receptor (*TSHR*), and thyroid peroxidase (*TPO*) (Antonica et al., 2012), which were all expressed by both murine and human thyroid gland-derived spheres (thyrospheres), but were not expressed by murine dermal fibroblasts or by murine or human submandibular salispheres (Nanduri et al., 2014; Pringle et al., 2016) (Figures S1A and S1B). Immunolabeling of both primary murine and human thyrospheres showed positive staining for NKX2.1, thyroglobulin, and T4, but not calcitonin, thus further confirming their tissue of origin (Figure 1C). During thyroid hormone production *in vivo*, iodinated thyroglobulin is converted into T3 and T4, which is secreted through the basal membrane into the bloodstream (Visser, 2018). However, in the primary murine organoids, basolateral T4 can be observed, which could be explained by the timing of fixation. In this staining, these primary organoids may have been secreting T4 into the environment at the time of fixation.

Next, to assess the presence of putative stem cells, the self-renewal potential of single thyrosphere-derived cells was evaluated by replating dissociated single cells in Matrigel and supplementing them with defined medium (Figure 1D, Video S1). After 1 week for murine and 2 weeks for human thyrospheres, the percentage of secondary structures, now termed organoids, was determined. For murine thyroid organoid formation, we tested two types of medium, TGM and TGM + WR, yielding similar potentials for self-renewal (Figures 1E and 1F), similar to what has been observed in the salivary gland (Maimets et al., 2016). However, to establish and propagate human organoid cultures, both Wnt and R-spondin1 were additionally required (Figures 1E and 1G). It should be noted that, while both murine and human cultures displayed some impurities after isolation (Figure 1B), likely consisting of red blood cells and fibroblasts, this debris disappeared during passaging, and only thyroid gland-derived cells remained (Figure 1E). Interestingly, comparing human- and murine-derived cells, a higher organoid-forming efficiency is displayed by the murine-derived culture. This may be due to interspecies differences in the number of cells capable of forming organoids with this specific culture medium. Similar differences have been observed in the salivary gland (Maimets et al., 2016; Pringle et al., 2016).

Video S1 Live-cell imaging of murine thyroid gland organoid during culturing

NKX2.1 co-localized with PAX8 in both murine and human thyroid organoids, while both organoid cultures also expressed sodium iodide symporter protein (NIS, also known as SLC5A5) necessary to produce thyroid hormone (Figure 1H, negative controls Figure S1C), confirming both thyroid phenotype and functionality. The majority of both murine and human organoids were found to express *NKX2.1*, *PAX8*, and *NIS* (Figure 1I). Moreover, organoids from passage 5 continued to express these genes, indicating that the cells maintained the phenotype from their tissue of origin during passaging (Figures S1D and S1E). Regardless of the *NKX2*.1 and *PAX8* co-expression, a variability can be seen in the expression of these two genes. It has previously been shown that such variations may be the result of cell-to-cell differences in gene expression, caused by factors such as a differing amount of transcription factors or the stage of the cell in the cell cycle (das Neves et al., 2010; Stewart-Ornstein et al., 2012). More recently, similar results have been demonstrated in the zebrafish thyroid gland (Gillotay et al., 2020). Furthermore, both murine and human organoids demonstrated the expression of the tight-junction marker ZO-1, indicating that these organoids are able to maintain and/or redevelop thyroid epithelium integrity (Figures 1H, S1D, and S1E).

We continued by analyzing the cell-cycle status of both murine and human thyroid organoids. After 4 days in culture, 13.5 ± 0.7% of the murine cells were in the S phase, while 16.0 ± 0.6% of the cells were in the G2 phase. No difference was seen in the S and G2 phases after 5 days in culture. However, after 7 days, a decrease was seen in the percentage of cells in the S and G2 phases (Figure S1F). Similarly, after 7 days of culture, 3.5 ± 0.7% of the human cells were in the S phase, while 14.0 ± 1.9% of the cells were in the G2 phase. After 21 days, no difference was seen in the percentage of cells in neither the S phase nor the G2 phase (Figure S1F). These data suggest a reduced proliferation potential in the murine organoids after prolonged culturing, while the human organoids maintain their proliferative capacity.

An important characteristic of stem cells is their ability to divide asymmetrically. This division can occur through random or non-random segregation (Conboy et al., 2007). By performing dual-pulse labeling, this DNA template segregation can be quantified through flow cytometry. Therefore, we used 5-ethynyl-2′-deoxyuridine (EdU) and bromodeoxyuridine (BrdU) to distinguish symmetrically and asymmetrically dividing cells. First, we assessed the percentage of dividing cells after 24 h using BrdU and Hoechst. Cells that have incorporated BrdU will not be able to bind Hoechst, indicating they have undergone division (Mozdziak et al., 2000). Flow cytometric analysis demonstrated that, after 24 h, almost half of the cells had incorporated BrdU, which was shown by a separate population lower in Hoechst labeling (Figure S1G). We then set up a gating strategy (Figures S1H1–S1H4), with negative controls (Figures S1H1 and S1H2) and singly labeled cells (Figures S1H3 and S1H4). Next, we performed double labeling by initially labeling the cells with EdU for the first 24 h, followed by another labeling with BrdU for another 24 h. Daughter cells that incorporated both EdU and BrdU had undergone random segregation and were most likely not stem cells. In contrast, the BrdU-labeled cells would have undergone non-random segregation and were potentially stem cells. Flow cytometric analysis indeed demonstrated that 15.8% of cells were BrdU positive (Figure 1J). Interestingly, this is in line with the organoid-forming efficiency of murine thyroid organoids in passage 3 in TGM + WR (Figure 1E) and may potentially indicate the number of stem cells. Additional staining for the stem cell marker SOX2 and proliferation marker Ki67 in murine organoids indeed demonstrated a specific subset of cells that were positive for both SOX2 and Ki67, indicative of proliferating putative stem cells (Figure S1I).

Culturing with Wnt and R-spondin1 did not induce differences in mRNA expression levels of thyroid differentiation markers in murine organoids for 5 passages (Figure S2A). However, flow cytometric analysis showed the presence of several stem cell enrichment markers (CD24/CD29 double positive, CD44, Sca-1, CD133, and EpCAM) after prolonged passaging (Figure S2B), further suggesting that these cultures seem to be stable and may contain putative stem cells, although a distinctive (thyroid) stem cell marker was not identified. Similar results have been shown in the salivary gland (Nanduri et al., 2014), where after several passages the organoids demonstrated an increase in stem cell enrichment markers. However, because these stem cell enrichment markers may occur in different subsets, each with its own self-renewal and differentiation potential, cells that express these enrichment markers are not necessarily all stem cells. Nonetheless, to shed more light on the putative stem cell population in the thyroid gland organoids after prolonged passaging, we performed staining for SOX2 and NKX2.1 at passages 5 and 10 in murine organoids. This showed that several cells are positive for either SOX2 or NKX2.1, which is more pronounced in passage 10 organoids than in passage 5, indicating that during prolonged passaging the number of undifferentiated (stem) cells seems to increase (Figure S2C). The thyroid gland organoids demonstrated almost homogeneous expression of similar stem cell enrichment markers, accompanied by a much lower organoid-forming potential, indicating that a specific combination of markers defining the thyroid stem cells has not been found yet. Nonetheless, high proliferation rates, as seen in Figure S1F, were reflected at the transcriptomic level by proliferation markers *Ki67* and *PCNA*, which showed significantly increased expression between human primary tissue-derived cells, early passage (passage 0–2) organoids, and later passage (passage 3–4) organoids (Figure S2D). A similar trend was observed after Ki67 and thyroglobulin co-immunofluorescence staining. Human organoids demonstrated an increase in the number of proliferating cells not expressing thyroglobulin, comparing passage 2 (3.8 ± 0.5%) and passage 5 (10.4 ± 2.5%) organoids (Figure S2E). Moreover, we observed an increased cell-cycle activity, as indicated by the elevated gene expression levels of several cyclin genes from early passages to later passages (Figure S2F, Table S1).

### *In Vitro* Maturation and Stemness of Thyroid Gland Organoids

**Figure 2:**
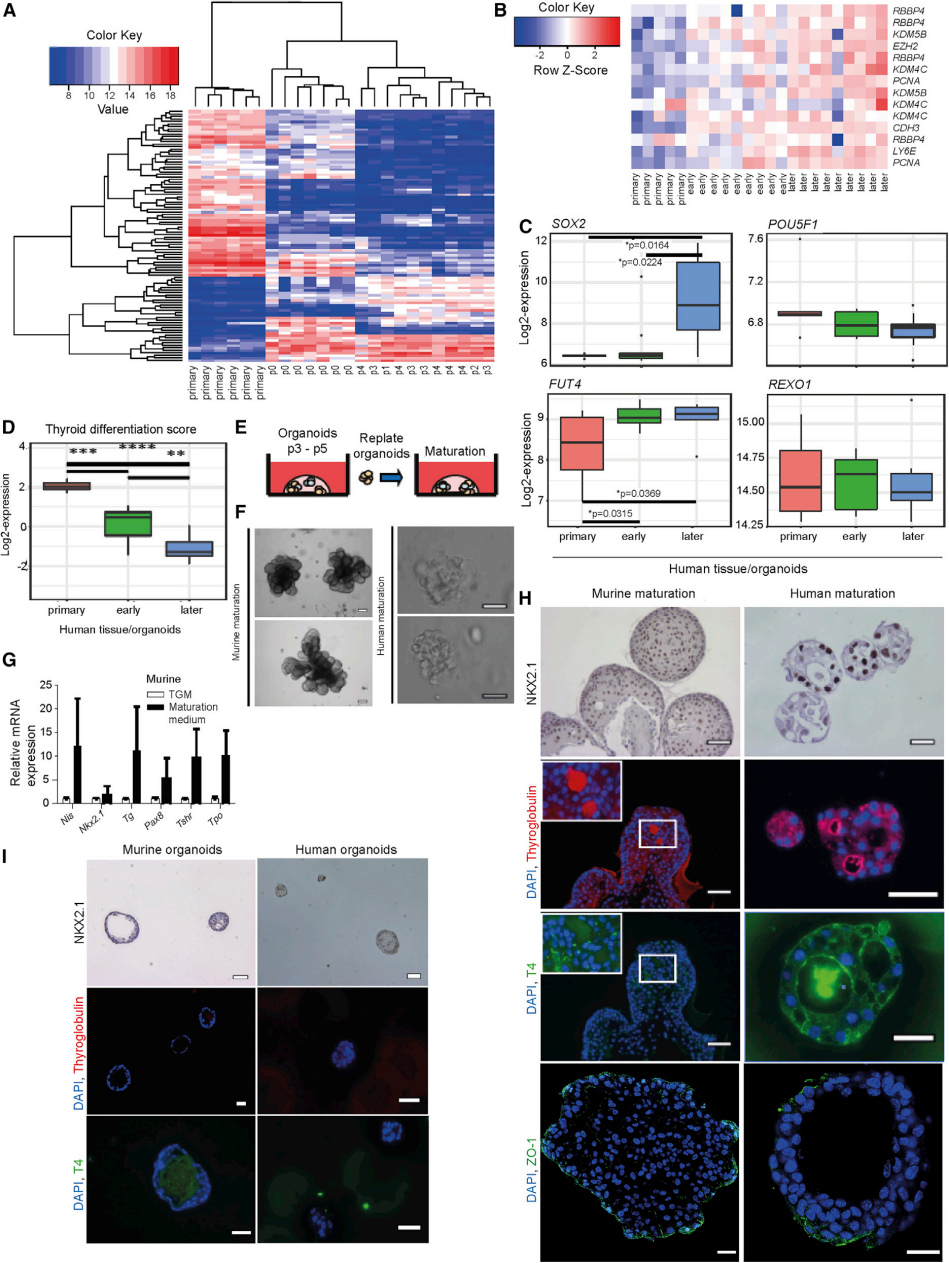
*In Vitro* Maturation and Stemness of Thyroid Gland Organoids (A) Global mRNA expression analyzed in primary human thyroid gland tissue-derived cells and organoids using SurePrint G3 Human Gene Expression 8×60K microarrays. The top 100 genes with the highest variance between all samples were subjected to unsupervised hierarchical clustering. (B) Heatmap showing genes with significantly increased expression (p < 0.05 with the use of two-sided Mann-Whitney test) from primary human thyroid gland tissue-derived cells to early (passage 0–2) and later (passage 3–4) organoids. (C) Boxplots with log2-expression values of stemness markers *SOX2*, *FUT4*, *POUSF1*, and *REXO1* in primary human thyroid tissue-derived cells (red, n = 5 independent donor biopsies) and early (passage 0–2; green, n = 9 independent donor biopsies) and later (passage 3–4; blue, n = 9 independent donor biopsies) passage organoids, two-sided Mann-Whitney test. (D) Comparison of thyroid differentiation score (Cancer Genome Atlas Research Network, 2014; Landa et al., 2016) values in primary human thyroid gland tissues (n = 5 independent donor biopsies) and early (passages 0–2, n = 9 independent donor biopsies) and later (passages 3–4, n = 9 independent donor biopsies) passage organoids, with the use of two-sided Mann-Whitney test. ^∗∗^p < 0.005; ^∗∗∗^p < 0.0005; ^∗∗∗∗^p < 0.00005, (E) Schematic representation of both murine and human thyroid organoid maturation protocol. Thyroid organoids were cultured for 3–5 passages using the self-renewal protocol, replated as organoids in Matrigel, and incubated in thyroid maturation medium. (F) Mature murine thyroid gland organoids and mature human thyroid gland organoids after 7 days in thyroid maturation medium. Scale bars, 50 μm. Pictures were cropped to show representative organoids. (G) qPCR for thyroid-specific markers showed that all thyroid-specific markers were expressed by the isolated murine cells after differentiation; a non-significant upward trend was present (n = 4 biological replicates) with the use of a two-sided Student t test. (H) NKX2.1, thyroglobulin, T4, and ZO-1 staining of mature murine and human thyroid gland organoids show a nuclear staining for NKX2.1, tight-junction staining by ZO-1, and positive follicle-like staining for thyroglobulin and T4. Scale bars, 50 μm for murine organoids and 25 μm for human organoids. Scale bars for ZO-1, 20 μm. Pictures were cropped to show representative organoids. (I) NKX2.1, thyroglobulin, and T4 staining of passage 4 murine thyroid gland organoids (n = 3 biological replicates) and passage 3 human thyroid gland organoids (n = 3 biological replicates) cultured in expansion medium. Scale bars, 50 μm. Pictures were cropped to show representative organoids. See also Figures S2 and S3 and Tables S1 and S2.

Next, we assessed the cell characteristics of primary tissue-derived cells and early (passage 0–2) and later (passages 3–4) organoid passages at the transcriptomic level by performing a microarray. Thyroid samples were separated into two main clusters, with the first cluster comprising all primary thyroid tissues of origin and the second cluster containing organoid samples only. The organoid-specific cluster was further divided into two subclusters, separating passage 0–2 samples from the remaining passage 3–4 samples. Regarding global gene expression, primary tissue-derived cells clearly differed from early and later passaged organoids, as shown by unsupervised hierarchical clustering and principal component analysis (Figures 2A and S3A). Further, we identified a set of genes that showed the highest significant increase in expression between the primary tissue-derived cells and the early passage organoids, and between early and later passage organoids (Figure 2B). These included mostly genes involved in histone binding and modification (*RBBP4*, *KDM5B*, *KDM4C*), survival (*LY6E*), cell adhesion (cadherin, *CDH3*), and proliferation (*PCNA*), potentially indicating enrichment of more primitive cells.

We continued by assessing the expression levels of the selected stem cell markers *SOX2*, *POU5F1*, *FUT4*, and *REXO1* (Ma et al., 2014) from the microarray. Whereas there was no difference between primary tissue-derived cells and early or later passage organoids for the genes *POU5F1* and *REXO1*, we detected a significant increase in *SOX2* from early to later organoid passages and of *FUT4* from primary tissue-derived cells to early organoid passages (Figure 2C), confirming enrichment for more primitive cells.

Our gene array analysis suggests an upregulation of genes expressed by more primitive cells upon passaging. This finding is supported by the observation that, during culture, 14 of 16 specific thyroid differentiation markers were decreased in expression (Figure S3B, Table S2). We summed the expression levels of these 16 genes involved in iodine metabolism and thyroid specification, which were highly correlated across our cohort, and produced a single metric, termed the thyroid differentiation score (TDS). Thyroid organoids showed a significantly decreased TDS compared with the tissue of origin, with later passages significantly more undifferentiated than early passages (Figure 2D). Subsequently, we investigated the maturation potential of the organoids using an adapted protocol from Kurmann et al. (Kurmann et al., 2015). We replated murine and human organoids in Matrigel after passages 3–5 of self-renewal and incubated them with thyroid maturation medium (Figure 2E). After 7 days, this resulted in a change in morphology, with both murine and human organoids forming thyroid gland-resembling structures (containing follicles) (Figure 2F), as well as expression of differentiation markers in the organoids (Figure 2G). Furthermore, these structures expressed the thyroid marker NKX2.1 (Figures 2H and S3C), and on average three thyroglobulin-filled colloids per structure were observed, with 8 ± 0.3% of the area being T4 positive in mice, whereas for humans four colloids per structure were thyroglobulin positive and 21 ± 1.2% of the area was T4 positive (Figure 2H). In addition, both the murine and the human organoids demonstrated expression of ZO-1, indicating that these organoids exhibited polarization upon maturation and preserved their epithelial integrity (Figure 2H). In contrast, none of the murine or human organoids cultured in expansion medium produced thyroglobulin or T4 (Figure 2I). Interestingly, comparing non-matured murine (Figure S1I) and non-matured human (Figure S2E) cells to their matured version, an increase in the number of Ki67-positive cells could be seen, indicating that maturation induction does not inhibit proliferation (Figure S3D). These results indicate that, although dedifferentiation occurs, the organoids are still able to differentiate to the major functional cell types of the thyroid gland. Moreover, the cumulative data obtained so far demonstrate that murine and human thyroid-derived cells are able to form organoids capable of *in vitro* self-renewal and differentiation.

### *In Vivo* Generation of Functional Thyroid Tissue

**Figure 3:**
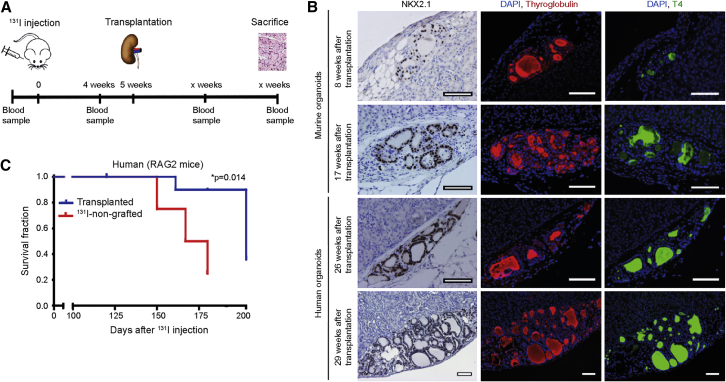
*In Vivo* Generation of Functional Thyroid Tissue in a Hypothyroid Mouse Model (A) Schematic representation of *in vivo* hypothyroid mouse model. On day 0, mice were injected with ^131^I. After 4 weeks, blood samples were taken for FT4 measurements to confirm hypothyroidism. Five weeks after ^131^I injection, mice were (xeno-)transplanted underneath the kidney capsule with dispersed murine or human organoid-derived cells from passage 3. After several time points, the mice were sacrificed for histological assessment. (B) NKX2.1, thyroglobulin, and T4 staining of thyroid follicles 8 and 17 weeks after transplantation of murine organoid-derived cells and 26 and 29 weeks after transplantation of human organoid-derived cells. Over time, larger thyroid structures were observed after both murine (17 weeks) and human (29 weeks) thyroid cell transplantation. Scale bars, 100 μm. (C) Survival in days after ^131^I injection. p = 0.014 with the use of log-rank test. See also Figure S4.

To study the generative capacity of the organoids, we transplanted dispersed murine and human thyroid organoids into a hypothyroid mouse model. By injecting radioactive iodine (^131^I), we induced hypothyroidism, which was confirmed by measuring free T4 (active form of T4) serum levels before and after ^131^I ablation (Figure 3A). Four weeks after ^131^I injection, free T4 levels declined to the detection limit of the assay (Figure S4A). Furthermore, in the non-ablated control mice, we found thyroid-specific follicles, as expected (Figure S4B), whereas 3–5 months after ^131^I ablation only thyroid gland remnants remained (Figure S4C).

Five weeks after ^131^I ablation, we transplanted 600,000 dispersed murine or human organoid-derived cells from passage 2 underneath the kidney capsule. Eight weeks later, murine thyroid gland cells formed follicular structures expressing NKX2.1 (56 ± 2.6%), thyroglobulin (35 ± 1.5% of area), and T4 (10 ± 0.3% of area) (Figure 3B). Both size and expression of NKX2.1 (75 ± 3.8%), thyroglobulin (71 ± 4.9% of area), and T4 (33 ± 2.4% of area) in the follicular structures were greatly increased 17 weeks after transplantation (Figure 3B). Similarly, after 26 weeks human thyroid gland cells formed follicular structures expressing NKX2.1 (76 ± 4.1%), thyroglobulin (20 ± 2.0% of area), and T4 (24 ± 1.5% of area). Although the expression of NKX2.1 (71 ± 11.9%) remained stable at 29 weeks post-transplantation, both thyroglobulin (86 ± 11.3% of area) and T4 (30 ± 9.5% of area) expression increased, indicating that the newly generated thyroid-derived structures had increased in size (Figure 3B). Furthermore, the presence of human nuclei indicated the clear xeno-engraftment of the human-derived cells (Figure S4D). Follicular structures were present at multiple locations of the kidney capsule (Figure S4E). Albeit very modestly, the levels of free T4 also increased with time, probably due to the limited number of cells injected and the limited number of blood vessels grown into the xenograft (Figures S4F and S4G). Moreover, animals injected with human cells that showed viable post-transplantation follicular tissue exhibited a prolonged survival compared with the ^131^I-treated sham-transplanted animals (Figure 3C). Together, these data show that primary thyroid gland-derived cells can self-renew into organoids and mature *in vitro* and *in vivo* into functional thyroid gland follicles, which produce free T4.

### Lack of Thyroid Tumor Markers in Thyroid Organoids

**Figure 4:**
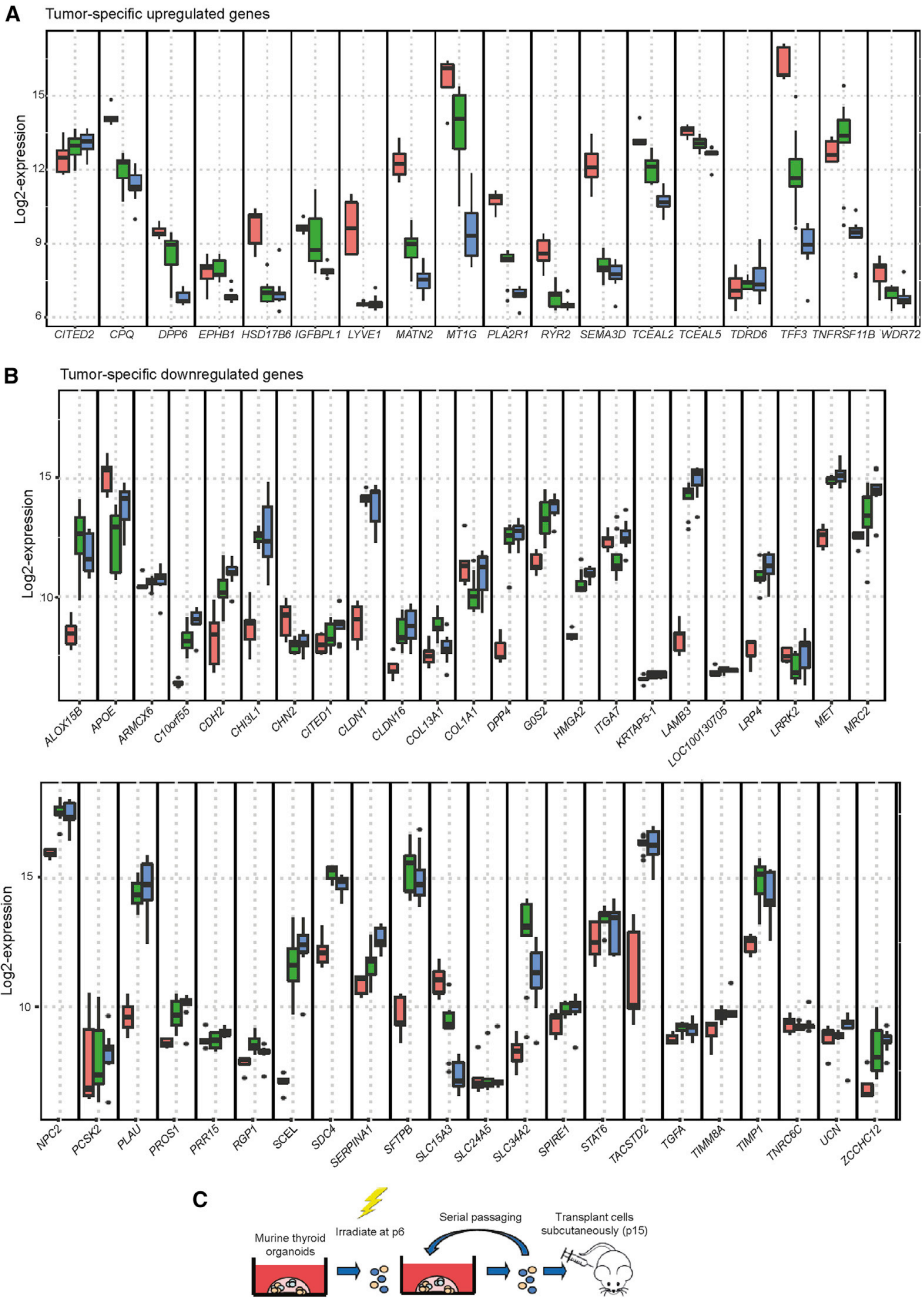
Thyroid Tumor Markers and Subcutaneous Transplantation of (Irradiated) Murine Thyroid Cells (A) Boxplots with log2-expression values of tumor-specific upregulated gene behavior in primary thyroid tissues (red, n = 5 independent donor biopsies) to the early (passage 0–2; green, n = 9 independent donor biopsies) and later (passage 3–4; blue, n = 9 independent donor biopsies) passage organoids. See Table S3 for p values, with the use of two-sided Mann-Whitney test. (B) Boxplots with log2-expression values of tumor-specific downregulated gene behavior in primary thyroid tissues (red, n = 5 independent donor biopsies) to the early (passage 0–2; green, n = 9 independent donor biopsies) and later (passage 3–4; blue, n = 9 independent donor biopsies) passage organoids. See Table S3 for p values, with the use of two-sided Mann-Whitney test. (C) Schematic representation of a subcutaneous *in vivo* mouse model to test the ability of non-irradiated and irradiated organoids to form tumor-like tissue. See also Table S3.

Increased proliferation and cyclin activity are also hallmarks of cancer cells (Chai et al., 2016a), thus we aimed to characterize the expression of genes that have previously been shown to be up- or downregulated in thyroid cancer (Agrawal et al., 2014; Chai et al., 2016b; Costa et al., 2015; Wang et al., 2017). Interestingly, 72% of genes described as upregulated in thyroid cancer were downregulated in early passage (passage 0–2) organoids compared with primary tissue-derived cells, while some of these genes displayed further downregulation in later passage (passage 3–4) organoids (Figure 4A, Table S3). Moreover, 71% of genes reported to be downregulated in thyroid cancer showed upregulation in early passage organoids, and the majority of these remained upregulated or further increased in later passage organoids (Figure 4B, Table S3). These data indicate that tumor-related markers are not induced by prolonged culturing.

Next, to assess potential transformation of transplanted cells, we irradiated murine thyroid organoid-derived cells with 1 Gy of X-rays. We cultured these cells to passage 15 and transplanted them subcutaneously to test tumorigenic potential (Figure 4C). As a positive control, we used a human follicular thyroid cancer cell line (FTC-133). Seven weeks after subcutaneous transplantation of cells from the FTC-133 cell line, tumors developed in all control mice transplanted with FTC-133 (n = 6), which warranted sacrificing the animals (Figure S4H). After 1 year, we detected no macroscopic tumors in the other animals (0 tumors in 12 mice) as well as the control transplantation without irradiation (n = 6), during weekly inspection or after sacrificing, indicating that these cells were not able to form tumors even after irradiation and long-term culturing.

## Discussion

Here, we demonstrate that both murine and human cells of the thyroid gland can be isolated, expanded *in vitro*, and cultured long term. These cells are capable of self-renewal and *in vitro* differentiation, suggesting that these cells possess a proliferative capacity required for expansion. Furthermore, after transplantation of a limited number of cells, these organoids form fully functional hormone-producing thyroid follicles in hypothyroid mice.

Our study demonstrates a proof of principle for the potential application of thyroid-derived organoids containing putative adult stem/progenitor cells in the treatment of hypothyroidism, which requires further investigation, optimization, and safety assessments. We characterized proliferation, differentiation, and regenerative potential for murine and human thyroid gland cells at the single-cell level *in vitro* and their long-term capabilities to form a functional mini-organ *in vivo*. However, we did not find a specific stem cell marker for the thyroid stem cells. A stem cell-specific marker would aid in the purification of thyroid stem cells for clinical application. However, it cannot be excluded that our culturing medium induces quiescent differentiated cells to revert to a stem cell state, such as observed in the liver, in which cholangiocytes have been shown to revert to a liver stem cell state to proliferate into hepatocytes upon impairment of liver regeneration (Raven et al., 2017). Therefore, instead of surface markers, the ability of cells to regenerate tissue should be further investigated (Clevers and Watt, 2018). Conversely, it should also be noted that previous research has shown the ability of primary murine thyrocytes to proliferate and form 3D lumen-containing structures *in vitro* (Koumarianou et al., 2017), while differentiated thyroid follicular cells have been shown to proliferate (Kimura et al., 2001). These findings suggest that the cellular expansion we observed both *in vitro* and *in vivo* may potentially be supported by the proliferative capacity of tissue-specific cells that are committed to the thyroid lineage.

Two important aspects of organoid culture render this technology highly suitable for follow-up studies. First, organoids can be expanded while maintaining genetic stability (Sato et al., 2011), which in our case is suggested by the lack of tumor-like gene expression profiles. Although we did not observe an increase in tumorigenic characteristics of prolonged cultured organoids or tumor formation after subcutaneous transplantation of organoid-derived cells, this does not indicate that these cells have no tumorigenic potential entirely. Therefore, prior to clinical application, the safety of the potentially transplanted population should be thoroughly assessed, for example, by DNA sequencing. Second, primary cell banks from individuals being treated for thyroid cancer could be generated by cryogenically storing organoids, containing putative stem cells, as has been shown for, among others, bone marrow and adipose tissue (Harris, 2014).

Although we observed thyroid gland formation after transplantation of both murine and human organoids under the kidney capsule, we have demonstrated only limited improvement of systemic T4 levels. Antonica and colleagues (Antonica et al., 2012) have previously generated *in vitro* functional follicles by using ESCs in which *NKX2*.1 and *PAX8* are overexpressed through doxycycline induction. However, solely inducing *NKX2*.1 and *PAX8* did not result in 3D sphere formation, and thus recombinant human thyroid-stimulating hormone (TSH) was added to the cell culture. Four weeks after transplantation of these ESCs into hypothyroid mice, restoration of thyroid hormone levels was observed. Compared with our experimental procedure, such a difference in time to restoration may be due to a higher number of cells transplanted (600,000 cells versus 2.5–3 million cells). In addition, no exogenous TSH was added (Antonica et al., 2012) to our cell culture to stimulate folliculogenesis. *In vitro* treatment of the thyroid-derived cells containing putative stem/progenitor cells with TSH or TSH administration to hypothyroid mice with transplanted thyroid cells may improve engraftment and accelerate folliculogenesis. Another issue could be the absence of blood vessels observed in our tissue structures; culturing organoids with endothelial cells may facilitate engraftment, as has been shown in, for example, the kidney (Low et al., 2019). Further optimization of the transplantation, and subsequently higher blood levels of T4, may be achieved by increasing the number of cells transplanted and vascularizing the organoids in advance to enhance tissue regeneration and decrease the time required for engraftment.

Adult stem cells hold great therapeutic promise in regenerative medicine and are not hampered by the ethical and genetic risks of the use of ESCs (Antonica et al., 2012; Volarevic et al., 2018). Furthermore, the importance of developing a safe autologous adult tissue-derived stem cell therapy is indicated by the long-lasting irreversible hypothyroidism upon surgical removal of the thyroid gland for malignant or benign indications or as a result of autoimmune thyroiditis, warranting lifelong daily thyroid hormone replacement therapy. Moreover, in the past few years, the number of LT4 prescriptions has been increasing, with a growth of 23 million additional prescriptions in the United States, and 10 million in the United Kingdom, between 2007 and 2014 (Rodriguez-Gutierrez et al., 2017). It is obvious that children need perfect dose delivery to support neurological development and growth, but 10%–15% of the adult patients undergoing thyroid hormone replacement therapy also suffer from persistent complaints related to their treatment (Benvenga, 2013). Although thyroid hormone replacement therapy is cheap and widely available and generally used in the treatment of hypothyroidism, without doubt about its overall efficacy (Wiersinga, 2001), it goes together with a number of side effects in a substantial amount of the patients (Garber et al., 2013). For the patients suffering from severe side effects, there is an unmet need for the development of a stem cell-based therapy considering the large number of patients affected by hypothyroidism (Thyroid Cancer - Cancer Stat Facts, 2019). However, several other obstacles have to be tackled, such as a persistent autoimmunity, the period of thyroid hormone-suppressive therapy in the first phase of cancer treatment, and the security of organoids that harbor occult cancer cells. With all stem cell therapies, utmost caution must be taken to ensure there are no risks of transformation, recurrences, and overproduction of the transplant. Placing the transplant at an easily accessible location in the body, e.g., subcutaneously or intramuscularly, would allow easy removal of the transplant in case of improper functioning. Therefore, further investigation is needed before a potential therapy can compete with hormone replacement therapies in both efficacy and safety.

In conclusion, we present murine and human thyroid organoid cultures containing cells with proliferative potential capable of self-renewal and differentiation, which, when transplanted into hypothyroid mice, form functional hormone-producing thyroid follicles. This highlights the proof of principle that thyroid organoid-derived cells can form a new mini-organ.

## Experimental procedures

All animal work was approved by the animal testing Ethical Committee of the University of Groningen. Non-malignant human thyroid gland tissue was obtained from donors, after informed consent and IRB approval (Thyrostem Study/METc 215/101), who were scheduled for thyroid surgery. Murine and human thyroid gland tissue was mechanically and enzymatically digested using collagenase I and dispase, followed by seeding into 12-well plates. Immunostaining was performed using paraffin-embedded sections. For (xeno-)transplantation, 600,000 dispersed organoid-derived cells were transplanted underneath the kidney capsule of hypothyroid mice.

Additional detailed experimental procedures can be found in the Supplemental Experimental Procedures.

### Data and Code Availability

The microarray data have been deposited in ArrayExpression (https://www.ebi.ac.uk/arrayexpress/) and can be accessed through the accession number (E-MTAB-6274) or via the following URL: https://www.ebi.ac.uk/arrayexpress/experiments/E-MTAB-6274/.

## Author contributions

V.O., A.G., N.H., T.L., J.P., and R.P.C. designed the project; V.O., A.G., N.H., P.N., J.H., A.J., M.B., and H.F. performed experiments; K.U. performed the bioinformatics analysis; A.G., V.O., N.H., and P.N. analyzed data; V.O., A.G., N.H., P.N., K.U., J.H., T.L., J.P., and R.P.C wrote the manuscript.

## Supplemental information

Document S1. Supplemental Experimental Procedures, Figures S1–S4, and Tables S1–S3

Document S2. Article plus Supplemental information
