# Supplementary material for: Generation and Differentiation of Adult Tissue-Derived Human Thyroid Organoids
Source: Stem Cell Reports. 2021 Mar 11;16(4):913–25. doi: 10.1016/j.stemcr.2021.02.011 (PMC8072035; doi:10.1016/j.stemcr.2021.02.011)
Supplement: Document S1. Supplemental Experimental Procedures, Figures S1–S4, and Tables S1–S3 [file mmc1.pdf]

Stem Cell Reports, Volume *16*

Supplemental Information

Generation and Differentiation of Adult Tissue-Derived Human Thyroid Organoids

Vivian M.L. Ogundipe, Andries H. Groen, Nynke Hosper, Peter W.K. Nagle, Julia Hess, Hette Faber, Anne L. Jellema, Mirjam Baanstra, Thera P. Links, Kristian Unger, John T.M. Plukker, and Rob P. Coppes

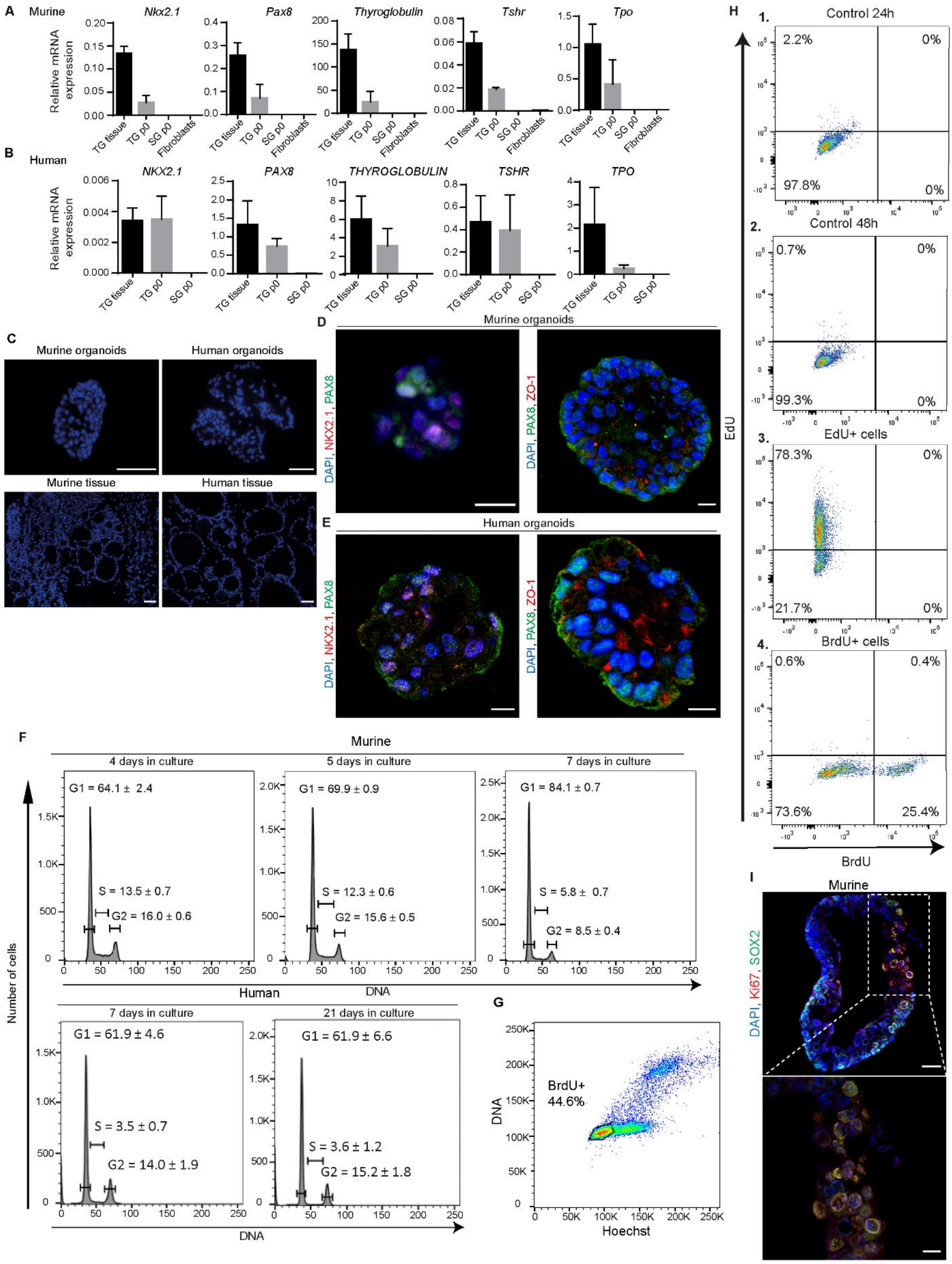

Figure S1. Characterization and culturing of murine and human thyroid organoids. Related to Figure 1.
A: qPCR for thyroid-specific markers showed that all thyroid specific markers (i.e., *NKX2.1*, *Pax8*, *Thyroglobulin*, *Tshr* and *Tpo*) were expressed by the isolated murine cells and not by primary dermal fibroblasts or primary spheres derived from submandibular salivary glands (n=4 biological replicates).
B: qPCR for thyroid specific markers showed that all thyroid specific markers (i.e., *NKX2.1*, *PAX8*, *THYROGLOBULIN*, *TSHR* and *TPO*) were expressed by the isolated human cells and not by primary spheres derived from submandibular salivary glands (n=4 biological replicates).

C: Negative controls of immunofluorescence assays for murine and human thyroid gland tissue and organoids. Scale bars = 50 µm.
D: Representative confocal images of co-immunofluorescent staining for NKX2.1 and PAX8, and PAX8 and ZO-1, respectively in murine organoids from passage 5. Scale bars = 20 µm.
E: Representative confocal images of co-immunofluorescent staining for NKX2.1 and PAX8, and PAX8 and ZO-1 in human organoids from passage 5. Scale bars = 20 µm.
F: Cell cycle analysis of passage 2 human (at day 7 and 21; n=3 biological replicates) and passage 3 (at day 4, 5, and 7; n=3 biological replicates) murine thyroid gland organoids to demonstrate the percentage of dividing cells during organoid culturing.
G: Flow cytometric analysis demonstrating percentage of cells that lack Hoechst and instead have incorporated BrdU after 24 hours.
H: Dual pulse labelling using EdU and BrdU. Flow cytometry plots demonstrate the percentage of cells containing EdU or BrdU or EdU and BrdU after one or two cell divisions, respectively.
I: Representative confocal images of immunofluorescent staining for Ki67 and SOX2 murine organoids from passage 2. Scale bars = 25 µm; insert scale bare = 10 µm.

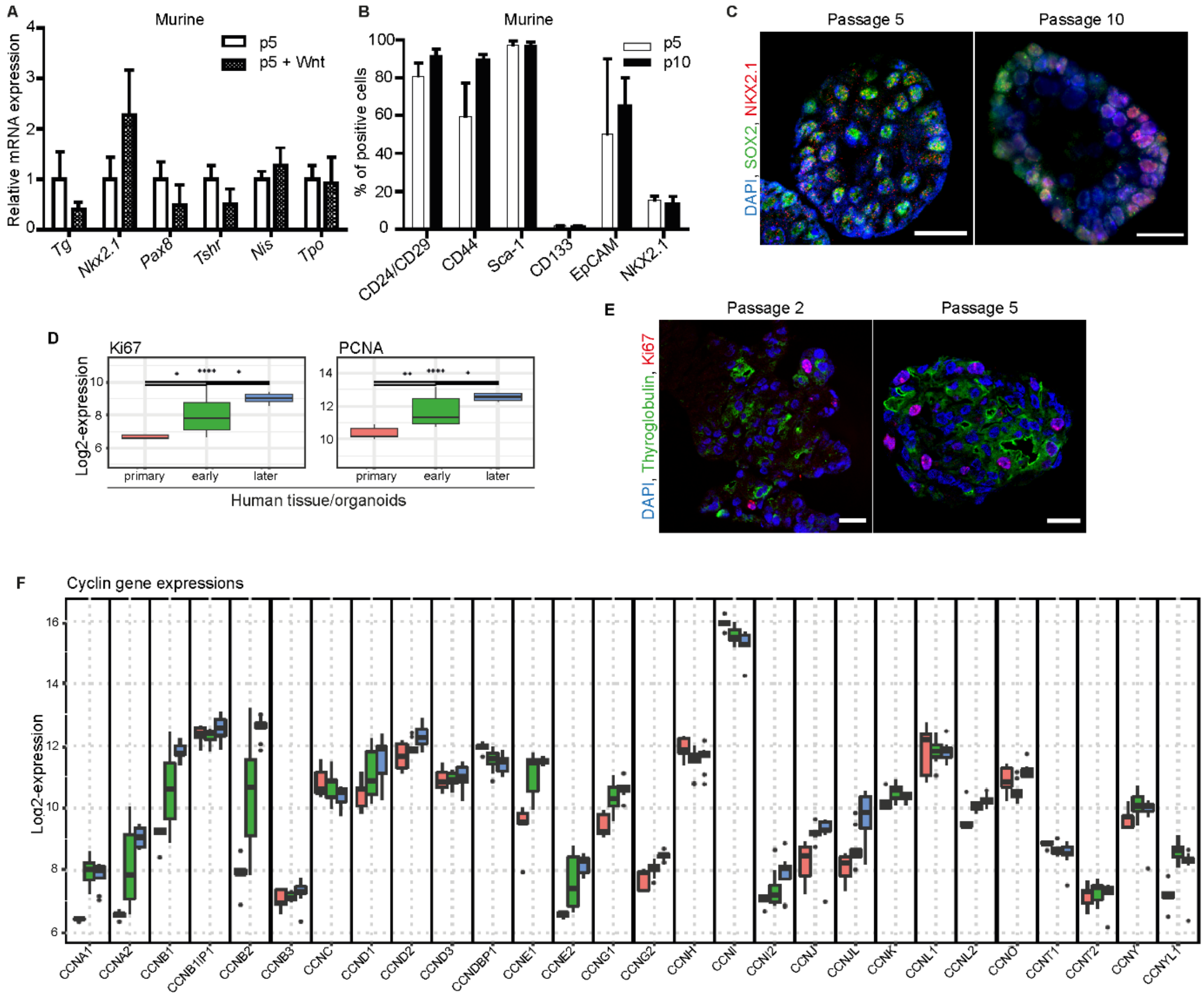

Figure S2. ***In vitro*** **maturation and proliferation of thyroid gland organoids. Related to Figure 2.**
A: qPCR for thyroid-specific markers showed that all thyroid-specific markers were expressed by the isolated murine cells when cultured with and without Wnt and R-spondin1 for 5 passages. No significant differences present in marker expression between cells grown in TGM and TGM+WR with the use of a two-sided student t-test (n=4 biological replicates).
B: Flow cytometric analysis showed early differentiation marker (NKX2.1), as well as several stem cell-like markers, to be present in murine thyroid organoids after 5 and 10 passage cultures in TGM.
C: Representative confocal images of co-immunofluorescence staining for SOX2 and NKX2.1in murine organoids from passage 5 and passage 10. Scale bar = 20 µm.
D: Boxplots with log2-expression values of proliferation markers Ki67 and PCNA in primary tissue (red, n=5 independent donor biopsies), early (passage 0-2; green, n=9 independent donor biopsies) and later (passage 3-4; blue, n=9 independent donor biopsies) passage organoids. * = p-value < 0.05; ** = p-value < 0.005; *** = p-value < 0.0005, with the use of two-sided Mann-Whitney test.
E: Representative confocal images of co-immunofluorescence staining for thyroglobulin and Ki67 in human organoids from passage 2 and passage 5. Scale bar = 20 µm.
F: Boxplots with log2-expression values of Cyclin genes in primary tissues (red, n=5 independent donor biopsies), early (passage 0-2; green, n=9 independent donor biopsies) and later (passage 3-4; blue, n=9 independent donor biopsies) passage organoids. In early passage organoids cyclin genes *CCNA1*, *CCNA2*, *CCNB1*, *CCNB2*, *CCNDBP1*, *CCNE1*, *CCNE2*, *CCNG1*, *CCNI*, *CCNJ*, *CCNY*, *CCNYL1* and in later passages *CCND2*, *CCNG2*, *CCNJL*, *CCNL2*, and *CCNO* were increased in expression. See **Table S2** for p-values, with the use of two-sided Mann-Whitney test.

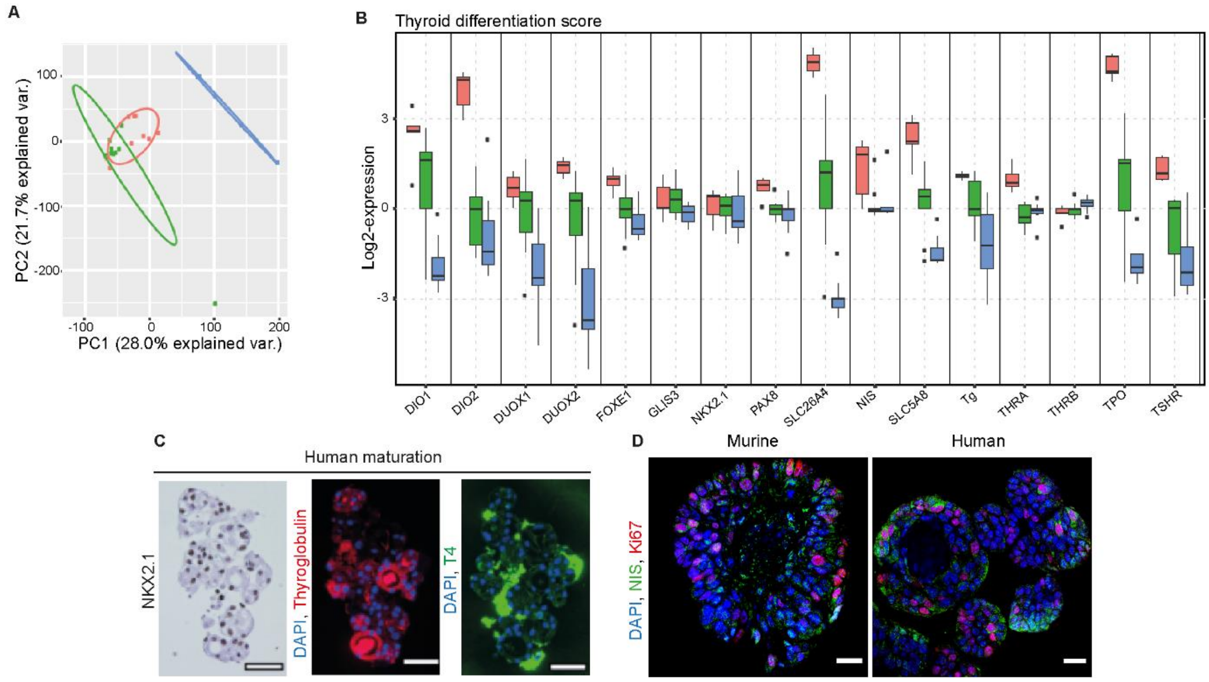

Figure S3. ***In vitro*** **differentiation of murine and human thyroid organoids. Related to Figure 2.**

A: Principal component analysis showing global gene expression in primary cultures (blue), which clearly differed from early (passage 0-2; red) and later (passage 3-4; green) passage organoids.

B: Comparison of 16 specific thyroid differentiation markers included in the thyroid differentiation score (see **Figure 2D**). During culturing we observed a decrease in 14 out of 16 specific thyroid differentiation markers in primary human thyroid gland tissues (red, n=5 independent donor biopsies) and early (passage 0-2; green, n=9 independent donor biopsies) and later (passage 3-4; blue, n=9 independent donor biopsies) passage organoids. See **Table S1** for p-values, with the use of two-sided Mann-Whitney test.

C: NKKX2-1, thyroglobulin and T4 staining of mature human thyroid gland organoids show a nuclear staining for NKX2-1 and positive follicle-like staining for thyroglobulin and T4. Scale bars = 25 µm. Pictures were cropped to show representative organoids.

D: Representative confocal images of co-immunofluorescence staining for NIS and Ki67 in murine and human matured organoids. Scale bar = 20 µm.

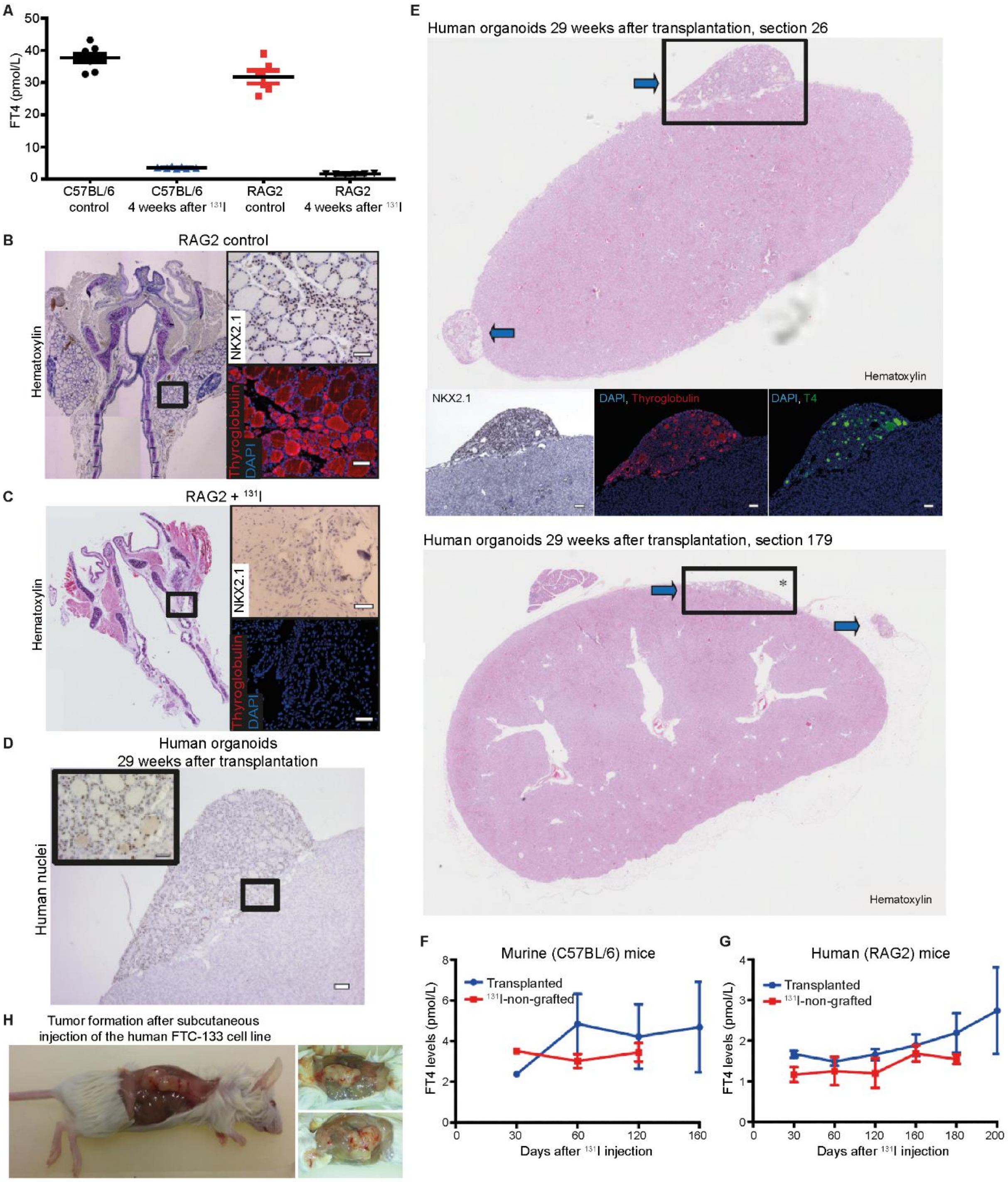

Figure S4. Thyroid remnants after ablation, and thyroid-like follicles. Related to Figure 3.

A: Four weeks after administration of 10 MBq ^{131}I, plasma levels of free T4 were significantly decreased from 37.7 (±3.6) pmol/L to 3.5 (±0.3) pmol/L (n=8) and from 32.8 (±2.8) pmol/L to 1.6 (±0.2) pmol/L (n=6), confirming the hypothyroid status of the mice.

B: Section of non-ablated neck area of RAG2 mouse. Normal thyroid tissue present, with a nuclear staining of NKX2.1 and a follicular pattern of the thyroglobulin staining. Scale bars = 50 µm.

C: Section of ^{131}I ablated neck area of RAG2 mouse. Only remnants of thyroid gland visible after ablation, without a nuclear staining of NKX2.1 and without any thyroglobulin staining, indicating successful ablation of thyroid gland by ^{131}I. Scale bars = 50 µm.

D: Positive nuclear staining with the human nuclei staining of the follicles, under the kidney capsule, confirming the human origin of the transplanted cells. Typical follicular structures were present at multiple locations underneath the kidney capsule, which expressed NKX2.1, thyroglobulin and T4. Scale bars = 100 µm and 50 µm of the insert.

E: Typical example of different sections (26 and 179) from a kidney of a RAG2 mouse 29 weeks after human thyroid organoid transplantation. Blue arrows indicate the multiple sites of follicular-like structures, which expressed NKX2.1, thyroglobulin and T4. See **Fig. 3B** for zoom-in of area indicated by *. Scale bars = 100 µm.
F: Mean FT4 levels at multiple time points after ^{131}I injection for both control mice (n=2) and mice transplanted with murine thyroid stem cells/organoids (n=9 biological replicates, mean ± SEM). C57BL/6 mice were used for mouse-to-mouse transplantations.
G: FT4 levels after multiple time points of ^{131}I injection for both control mice (n=3) and mice transplanted with human thyroid stem cells/organoids (n=7 biological replicates, mean ± SEM). For human-to-mouse transplantations immunosuppressed RAG2 mice were used.
H: Tumor formed after subcutaneous injection of human thyroid cancer FTC-133 cell line (n=6).

Supplementary Table 1. P-values of the change in cyclin genes. Related to Figure 2.

Gene	p-value primary vs. early	p-value early vs. later
CCNA1	0.001 (↑)	0.5457 (↓)
CCNA2	0.004 (↑)	0.13591 (↓)
CCNB1	0.01199 (↑)	0.06253 (↑)
CCNB1IP1	0.51848 (↓)	0.13591 (↑)
CCNB2	0.01199 (↑)	0.05031 (↑)
CCNB3	0.60639 (↑)	0.1615 (↑)
CCNC	0.6993 (↑)	0.06253 (↓)
CCND1	0.23976 (↑)	0.1615 (↑)
CCND2	0.2977 (↑)	0.03147 (↑)
CCND3	0.7972 (↑)	0.60481 (↑)
CCNDBP1	0.01898 (↓)	0.43628 (↓)
CCNE1	0.002 (↑)	0.48943 (↑)
CCNE2	0.002 (↑)	0.29733 (↑)
CCNG1	0.00699 (↑)	0.19025 (↑)
CCNG2	0.05994 (↑)	0.00049 (↑)
CCNH	0.08292 (↓)	0.25808 (↑)
CCNI	0.01199 (↓)	0.11349 (↓)
CCNI2	0.43756 (↑)	0.19025 (↑)
CCNJ	0.002 (↑)	0.22242 (↑)
CCNJL	0.05994 (↑)	0.01419 (↑)
CCNK	0.14685 (↑)	0.09391 (↓)
CCNL1	0.7972 (↓)	0.79617 (↓)
CCNL2	0.08292 (↑)	0.01419 (↑)
CCNO	0.14685 (↓)	0.00123 (↑)
CCNT1	0.08292 (↓)	0.43628 (↓)
CCNT2	0.36364 (↑)	0.43628 (↓)
CCNY	0.04196 (↑)	0.5457 (↓)
CCNYL1	0.001 (↑)	0.07701 (↓)

Supplementary Table 2. P-values of the change in thyroid differentiation score genes. Related to Figure 2.

Gene	p-value primary vs. early	p-value early vs. later
DIO1	0.05994 (↓)	0.00276 (↓)
DIO2	0.001 (↓)	0.11349 (↓)
DUOX1	0.18981 (↓)	0.01876 (↓)
DUOX2	0.00699 (↓)	0.00777 (↓)
FOXE1	0.01898 (↓)	0.25808 (↓)
GLIS3	1	0.05031 (↓)
NKX2-1	0.43756 (↓)	0.73044 (↑)
PAX8	0.01199 (↓)	0.73044 (↓)
SLC26A4	0.001 (↓)	0.00016 (↓)
SLC5A5	0.00699 (↓)	0.86331 (↓)
SLC5A8	0.002 (↓)	0.00777 (↓)
TG	0.04503 (↓)	0.02443 (↓)
THRA	0.001 (↓)	0.48943 (↑)
THRB	0.7972 (↑)	0.11349 (↑)
TPO	0.001 (↓)	0.00777 (↓)
TSHR	0.001 (↓)	0.22242 (↓)

Supplementary Table 3. P-values of the change in thyroid tumor-specific up- and downregulated genes. Related to Figure 4.

**Thyroid tumor-specific upregulated genes**		
**Gene**	**p-value primary vs. early**	**p-value primary vs. later**
CITED2	0.14685 (↑)	0.5457 (↑)
CPQ	0.001 (↓)	0.03147 (↓)
DPP6	0.01898 (↓)	0.00078 (↓)
EPHB1	1	0.00008 (↓)
HSD17B6	0.001 (↓)	0.79617 (↓)
IGFBPL1	0.43756 (↓)	0.01061 (↓)
LYVE1	0.001 (↓)	1
MATN2	0.001 (↓)	0.00123 (↓)
MT1G	0.04196 (↓)	0.00029 (↓)
PLA2R1	0.001 (↓)	0.00777 (↓)
RYR2	0.001 (↓)	0.1615 (↓)
SEMA3D	0.001 (↓)	0.1615 (↓)
TCEAL2	0.001 (↓)	0.00008 (↓)
TCEAL5	0.01898 (↓)	0.00705 (↓)
TDRD6	0.43756 (↑)	0.93143 (↓)
TFF3	0.001 (↓)	0.00016 (↓)
TNFRSF11B	0.23976 (↑)	0.00016 (↓)
WDR72	0.04196 (↓)	0.25808 (↓)
**Thyroid tumor-specific downregulated genes**		
**Gene**	**p-value primary vs. early**	**p-value primary vs. later**
ALOX15B	0.001 (↑)	0.1615 (↓)
APOE	0.001 (↓)	0.02443 (↑)
ARMCX6	0.51848 (↑)	0.25808 (↑)
C10orf55	0.001 (↑)	0.01419 (↑)

CDH2	0.004 (↓)	0.03998 (↑)
CHI3L1	0.001 (↑)	0.93143 (↓)
CHN2	0.00699 (↓)	0.34011 (↑)
CITED1	0.2977 (↑)	0.25808 (↑)
CLDN1	0.001 (↑)	0.73044 (↑)
CLDN16	0.004 (↑)	0.43628 (↑)
COL13A1	0.004 (↑)	0.00399 (↓)
COL1A1	0.02897 (↑)	0.07701 (↑)
DPP4	0.001 (↑)	0.38651 (↑)
G0S2	0.001 (↑)	0.30963 (↑)
HMGA2	0.001 (↑)	0.03998 (↑)
ITGA7	0.08292 (↓)	0.03998 (↑)
KRTAP5-1	0.05994 (↑)	1
LAMB3	0.001 (↑)	0.01876 (↑)
LOC100130705	0.2977 (↑)	0.66647 (↑)
LRP4	0.001 (↑)	0.07701 (↑)
LRRK2	0.08292 (↓)	0.09391 (↑)
MET	0.001 (↑)	0.1615 (↑)
MRC2	0.08292 (↑)	0.05031 (↑)
NPC2	0.001 (↑)	0.60481 (↓)
PCSK2	0.7972 (↑)	0.66647 (↑)
PLAU	0.001 (↑)	0.22242 (↑)
PROS1	0.001 (↑)	0.29733 (↑)
PRR15	0.7972 (↑)	0.03998 (↑)
RGP1	0.001 (↑)	0.06253 (↓)
SCEL	0.001 (↑)	0.29733 (↑)
SDC4	0.001 (↑)	0.02443 (↓)
SERPINA1	0.02897 (↑)	0.00123 (↑)
SFTPB	0.001 (↑)	0.43628 (↓)
SLC15A3	0.004 (↓)	0.00123 (↓)
SLC24A5	1	0.5457 (↑)
SLC34A2	0.002 (↑)	0.02443 (↓)
SPIRE1	0.08292 (↑)	0.60481 (↑)
STAT6	0.02897 (↑)	0.60481 (↓)
TACSTD2	0.00332 (↑)	0.96475 (↓)
TGFA	0.00699 (↑)	0.5457 (↓)
TIMM8A	0.004 (↑)	0.5457 (↑)
TIMP1	0.001 (↑)	0.13591 (↓)
TNRC6C	0.7972 (↑)	0.66647 (↓)
UCN	0.8981 (↑)	0.00399 (↑)
ZCCHC12	0.00699 (↓)	0.29733 (↑)

SUPPLEMENTAL EXPERIMENTAL PROCEDURES

Isolation of murine thyroid gland cells

All performed animal work was approved by the animal testing Ethical Committee of the University of Groningen. To limit the number of animals used for the experiments, the thyroid glands from 8-12-week-old female C57BL/6 mice (Harlan, The Netherlands), which were required for another research project, were dissected. Thyroid gland tissue derived from 3 mice were collected in one tube in Hank's Balanced Salt Solution (HBSS) containing 1% BSA and mechanically digested it using the GentleMACS dissociator (Miltenyi Biotech), followed by enzymatic digestion in HBSS/1%BSA buffer containing collagenase I (100U/ml; Gibco) and dispase (1,5U/ml; Gibco). All cells were seeded into one well of a 12-well plate in TGM consisting of DMEM-F12 medium (Gibco) containing 1% penicillin/streptomycin (Gibco), glutamax (2mM, Gibco), epidermal growth factor (20ng/ml, Sigma Aldrich), fibroblast growth factor-2 (20ng/ml, Peprotech) and 0.5% B27 supplement (Gibco).

Collection and isolation of human thyroid gland cells

Non-malignant human organoid thyroid gland tissue was obtained from donors age 19-80 during scheduled thyroid surgery (after informed consent and IRB approval (Thyrostem study / METc 215/101)). Apparently macroscopic normal thyroid tissue was dissected from the contralateral side of DTC patients, and from patients suffering from goitre, which has been considered a benign condition with low risk for malignancy. The thyroid gland was removed according the treatment plan as decided by the institutional multidisciplinary thyroid board. Furthermore, the biopsy spots were marked and the pathologist evaluated whether the tumor was multifocal, bilateral and whether there was tumor close to the biopsy. This cohort represents the patient group most eligible for stem cell transplantation following clinical translation.

Biopsies were collected under sterile conditions within 10 minutes after thyroidectomy and transported them in a Greiner tube in HBSS containing 1% BSA. Mechanical digestion using the GentleMACS dissociator was followed by enzymatic digestion in HBSS/1%BSA buffer containing collagenase I (100U/ml) and dispase (1,5U/ml). $8x10^5$ cells/ml were seeded into 1-4 wells of a 12-well plate in Basement Membrane Matrigel (Matrigel; BD Biosciences, 354234) with TGM+WR and several other growth factors consisting of DMEM-F12 medium containing 50% Wnt conditioned media, 10% R-spondin1 conditioned media, 1% penicillin/streptomycin, glutamax, epidermal growth factor, fibroblast growth factor-2, 0.5% B27 supplement, 1% Heparin Sodium Salt solution (Stemcell technologies), nicotinamide (10mM; Sigma Aldrich), A83-01 (500nM; Tocris bioscience) and noggin (25ng/ml; Peprotech).

In vitro self-renewal assay

One day after isolation, murine primary spheres (passage 0) were dissociated into single cells using 0.05% trypsin-EDTA (Gibco), counted and resuspended at $8x10^5$ cells/ml. 25µl of this cell suspension was added to 50µl Basement Membrane Matrigel in 12-well culture plates. Twenty minutes after seeding, we added medium supplemented with ROCK inhibitor (Y-27632; Sigma-Aldrich) to the gels. One week after seeding, the secondary spheres were counted and the percentage of sphere forming cells was calculated. To test the long-term self-renewal ability of the cells, we passaged these secondary spheres every week by first adding dispase (1 mg/ml) to the gels to release the spheres from them, then disrupted the spheres into single cells using 0.05% trypsin-EDTA, and then reseeding $2.7x10^5$ single cells/ml in Matrigel®.

Seven days after isolation and seeding, human primary spheres (passage 0) were released from the gels by adding dispase (1 mg/ml) to the gels; these spheres were dissociated into single cells using 0.05% trypsin-EDTA, counted and resuspended at $8x10^5$ cells/ml. 25µl of this cell suspension was added to 50µl Basement Membrane Matrigel in 12-well culture plates. Twenty minutes after seeding, medium supplemented with ROCK inhibitor was added to the gels. One week after seeding 0.5 ml of fresh media was added; two weeks after seeding secondary spheres were counted and the percentage of sphere forming cells was calculated. To test long term self-renewal ability of the cells, we passaged these secondary spheres every two weeks by first adding dispase (1 mg/ml) to the gels to release the spheres from the gels, then disrupting the spheres into single cells using 0.05% trypsin-EDTA and reseeding $2.7x10^5$ single cells/ml in Matrigel®.

The cell numbers which were seeded at the beginning of each passage and the number of organoids counted at the end of each passage were used to calculate the organoid forming efficiency for each passage, using the following formula, in which OFE = organoid forming efficiency (%):

$$\text{OFE (\%)} = \frac{number\ organoids\ counted\ at\ end\ of\ passage}{number\ of\ cells\ seeded\ at\ beginning\ of\ passage}\ x\ 100$$

***In vitro* cell cycle analysis**

Murine organoids in passage 6 and human organoids in passage 3 were harvested and dissociated into a single cell suspension. Cells were fixed in 70% ethanol, and incubated overnight at 4ºC. Cells were washed twice in PBS supplemented with 0.2% BSA and centrifuged at 400g for 5 min. The cell pellet was resuspended in PureLink RNase A (Invitrogen), and incubated for 30 min at 37ºC. Cells were resuspended in propidium Iodide (PI; 50µg/ml), and incubated for one hour at room temperature (RT).

Dual pulse labelling for asymmetric division

To assess the percentage of dividing cells, murine single cells were seeded in Matrigel® and incubated with BrdU (10µM; Sigma) for 24 hours at 37ºC. After 24 hours, the cells were washed and dispersed, followed by incubation in Hoechst 33342 (10µg/ml; Sigma) and Propidium Iodide (PI; 10µg/ml; Sigma) diluted in PBS/1%BSA for 2 hours in the dark on ice.

To analyze asymmetric division, after the *in vitro* self-renewal assay, at the beginning of a passage, murine cells were incubated with Edu (10µM; Sigma) for 24 hours at 37ºC followed by an incubation with BrdU (10µM; Sigma) for 24 hours at 37ºC in 5% CO_2. After incubation with BrdU, cells were dissociated into single cells, fixed with 70% ethanol, and incubated overnight at 4ºC. Cells were centrifuged at 400g for 5 min, and the cell pellet was vortexed. The pellet was resuspended in 2.5M hydrochloric acid fuming (MERCK) and incubated for 20 min at RT. Phosphate/citric acid buffer was added, and the cells were centrifuged. The pellet was washed twice in PBS supplemented with 0.1% Triton-X and 1% BSA. Click reaction was prepared in PBS using $CuSO_4$ (4 mM), Sulfo-Cy3 azide (20µM), and ascorbic acid (10 mM). The cells were incubated in the click reaction for 30 min in the dark at RT. Here after, the cells were incubated with mouse monoclonal anti-BrdU antibody [MoBU-1] (1:100; Thermo Fisher Scientific) for 30 min in the dark at RT, and incubated with goat anti-mouse conjugated with Alexa-647 (1:1000; Sigma) for 30 min in the dark at RT. Hoechst33342 (5µg/ml; Sigma) was added, and dual pulse labelling was analyzed using LSR-II (BD Biosciences).

***In vitro* differentiation assay**

To test the differentiation capacity of thyrospheres we performed an adjusted differentiation protocol based on Kurmann *et al* (Kurmann et al., 2015). Murine and human 1 or 2 week old thyrospheres were re-plated from passages 3-5 in a 24-well plate in growth factor reduced Matrigel, and incubated with thyroid maturation media consisting of DMEM F12 supplemented with Heparin Sodium Salt Solution (100ng/ml), IGF-1 (50ng/ml), fibroblast growth factor-2 (20ng/ml), fibroblast growth factor-10 (100ng/ml, Peprotech), 0.5% B27 supplement, insulin (5µg/ml, Sigma Aldrich), ITS (5µg/ml, Gibco), EGF(25ng/ml), Dexamethasone (50nM, Sigma Aldrich), and bTSH (100 mU/ml, Sigma Aldrich). On day 7, the organoids were collected for qPCR or embedded in paraffin for immunofluorescence.

Transplantation of thyroid cells into induced hypothyroid mice

For mouse-to-mouse transplantations, C57BL/6 mice (8-12-week-old females, Harlan, The Netherlands) were used. The mice were maintained under conventional conditions and fed them ad libitum. The Animal Ethical Committee of the University of Groningen approved all experimental procedures. To induce hypothyroidism, the mice were placed on a low-iodine diet (custom iodine deficient food, SAFE) for 8 days and were administered an intraperitoneal injection of 10 MBq of ^{131}I. Four weeks after administration of ^{131}I, the plasma levels of free T4 were analysed to confirm the hypothyroid status of the mice. One week later (5th week), the hypothyroid mice we anaesthetized using isoflurane/O2 inhalation and transplanted 5µl of dissociated murine thyroid gland organoids (passage 2; containing 1×10^6 cells) underneath the kidney capsule using a 28G needle syringe (Hamilton). Plasma levels of free T4 were analysed every 20-40 days after transplantation to assess the thyroid status of the mice. The kidneys were collected 8 and 17 weeks after transplantation and histology of the transplant was assessed.

For human-to-mouse transplantations the same protocols and procedures were used. However, in this case we used, immunosuppressed RAG2 mice containing a disruption of the recombination activating gene 2 (RAG2) and therefore failing to produce mature B or T lymphocytes. These were purchased from Jackson Lab (Maine, United States). These mice were transplanted with 5µl of dissociated human thyroid gland organoids (passage 2; containing 1×10^6 cells) and the kidneys were collected 26 and 29 weeks after transplantation and histology of the transplant was assessed.

Subcutaneous transplantation of murine thyroid cells

We used C57BL/6 mice (8-12-week-old females) to test the tumorigenic potential of cultured and irradiated murine thyroid gland cells, which were transplanted subcutaneously with Matrigel. The mice were anesthetized by isoflurane/O2 inhalation and subcutaneously transplanted into them 600.000 murine thyroid gland cells (passage

15; n=6), thyroid gland cells irradiated with 1 Gy of X-rays (passage 6; n=6), all using a 28 G needle syringe (Hamilton). As a positive control, 600.000 cells of a human follicular thyroid cancer cell line (FTC-133) which is known to form tumors after subcutaneous transplantation were transplanted (n=6). Every week until one year after transplantation the subcutaneous injection sides were checked for tumor formation. The histology of the transplant (if present) was analyzed.

Microarray-based gene expression analysis

Total RNA was extracted from cell culture and primary tissue samples using the RNeasy Mini Kit (Qiagen, Valencia, CA, USA) according to manufacturer's instructions. The TissueLyser (Qiagen) was used to disrupt and homogenize primary tissue samples. RNA integrity was assessed using the 2100 Bioanalyzer (Agilent Technologies, Santa Clara, CA, USA) in combination with the RNA 6000 Nano Kit (Agilent Technologies): all samples showed a RIN of > 7. Global gene expression profiling was performed using SurePrint G3 Human Gene Expression 8x60k microarrays (v3, AMADID 72363, Agilent Technologies) according to the manufacturer's protocol with an input of 50 ng of total RNA (one-color Low Input Quick Amp Labeling Kit, Agilent Technologies). Hybridized microarrays were scanned with a G2505C Sure Scan Microarray Scanner (Agilent Technologies) and raw data were extracted with Feature Extraction 10.7 software (Agilent Technologies). Data quality assessment, preprocessing, normalization, and differential expression analyses were conducted using the R Bioconductor packages limma (Ritchie, Phipson et al. 2015) and Agi4x44PreProcess as described in Hess *et al*. 2017 (Hess et al., 2017). Up on acceptance of the manuscript a GEO accession number will be applied for.

The continuous thyroid differentiation score (TDS) ranked the samples according to RNA expression levels within selected set of 16 thyroid function genes. TDS was originally developed to quantify relationships between thyroid differentiation and diverse genetic or epigenetic events. The Log2-normalized RSEM values were first centered at the median across samples, yielding Log2(Fold Change), and then summed across the 16 genes for each sample: TDS = Mean of Log2(Fold Change) across 16 genes, in primary human thyroid gland tissues (n=5) and early (passages 0-2, n=9) and late (passages 3-4, n=9) passage organoids. Horizontal lines within each box of all the boxplots represent median values, box heights symbolize the interquartile range (IQR) (IQR = Q3–Q1); Q3, whiskers represent values up to 1.5 times IQR greater than Q3 (top: Q3 + 1.5 × IQR) or smaller than Q1 (bottom: Q1 – 1.5 × IQR). * = p-value < 0.05, ** = p-value < 0.005; *** = p-value < 0.0005; **** = p-value < 0.00005, two-sided Mann-Whitney test.

RNA isolation and qRT-PCR

Total RNA from thyroid gland cells was isolated using the RNeasy Mini Kit (Qiagen) according to the manufacturer's protocol. Next, total RNA was reverse-transcribed using the Superscript II kit (Invitrogen) according to the manufacturer's instructions. Quantitative PCR (qPCR) was performed in triplicate using Power SybrGreen mix and an IQ5 Real-Time PCR System (Biorad). Relative mRNA levels were calculated as $2^{-\Delta CT}$, in which ΔCT is the CT value of target gene – CT value of the reference gene YWHAZ. The following primers were used:

Murine samples:

*Ywhaz*_for, 5'- TTACTTGGCCGAGGTTGCT -3';

*Ywhaz*_rev, 5'- TGCTGTGACTGGTCCACAAT -3';

*Nkx2.1(Ttf-1)*_for, 5'- CGCCTTACCAGGACACCAT -3';

*Nkx2.1(Ttf-1)*_rev, 5'- CCCATGCCACTCATATTCAT -3';

*Pax8*_for, 5'- CAGCCTGCTGAGTTCTCCAT -3';

*Pax8*_rev, 5'- CTGTCTCAGGCCAAGTCCTC -3';

*Tshr*_for, 5'- GTCTGCCCAATATTTCCAGGATCTA -3';

*Tshr*_rev, 5'- GCTCTGTCAAGGCATCAGGGT -3';

*Thyroglobulin*_for, 5'- AGGACCCGTGTGGTAGG -3';

*Thyroglobulin*_rev, 5'- CTGACCCAGAGAATGGCAGT -3';

*Tpo*_for, 5'- ACAGTCACAGTTCTCCACGGATG -3';

Tpo _rev, 5'- ATCTCTATTGTTGCACGCCCC -3';

Human samples:

*YWHAZ*_for, 5'- TTACTTGGCCGAGGTTGCT -3';

*YWHAZ*_rev, 5'- TGCTGTGACTGGTCCACAAT -3';

*NKX2.1 (TTF-1)*_for, 5'- TCATTTGTTGGCGACTGG -3';

*NKX2.1 (TTF-1)*_rev, 5'- TGCTTTGGACTCATCGACAT -3';

*PAX8*_for, 5'- GAGGTGAGGTGAGGTGTGGTGTGC -3';

*PAX8*_rev, 5'- GGTTTCCTGCGATTCTGC -3';

TSHR _for, 5'- AAAGAGCTCCCCCTCCTAAA -3';

TSHR _rev, 5'- TTGGTCAGGTCAGGGAACAT -3';

THYROGLOBULIN _for, 5'- AAGCCTCTGCAATGTGCTC -3';

THYROGLOBULIN _rev, 5'- GGACATAGCCTGGGCTGAC -3';

TPO _for, 5'- CAGCCCATGGACATTACTCC -3';

TPO _rev, 5'- TTGCAAGAAGGCCTCGTATT -3';

Immunofluorescence and immunohistochemistry

Spheres/organoids were fixed in 4% paraformaldehyde (Electron microscopy sciences) for 30 minutes and explanted kidneys were fixed for 24h. Fixed spheres, organoids and kidney tissue was processed for paraffin embedding and cut into 3.5-4 µm thick sections. Sections were dewaxed and incubated with rabbit monoclonal NKX2-1 [EP1584Y] (1:100; Abcam), Anti-Human Nucleoli antibody [NM95] (1:200; Abcam), polyclonal rabbit anti-human Thyroglobulin (1:8000; DAKO), polyclonal rabbit anti thyroxine (1:100; T4; Cloud-Clone Corp), mouse monoclonal PAX-8 [MRQ-50] (1:100; Cell Marque), and rabbit polyclonal sodium iodide symporter (1:50; Proteintech) overnight at 4ºC. Visualization for bright-field microscopy was performed by incubation with biotinylated polyclonal swine anti-rabbit immunoglobulins (1:300; DAKO) for 1-2 hours at RT, followed by incubation with VECTASTAIN Elite ABC HRP Kit (Vector Laboratories) for 1 hour at RT, and the diaminobenzidine (SIGMAFAST™ 3,3′-Diaminobenzidine tablets, Sigma Aldrich) chromogen. Nuclear counterstaining was performed with hematoxylin. Sections incubated with Thyroglobulin were incubated with goat anti-rabbit IgG (Alexa Fluor® 594 conjugate; 1:1000; Thermofischer) and sections stained for T4 with goat anti-rabbit IgG (Alexa Fluor® 488 conjugate; 1:1000; Thermofisher) for 1 hour at RT. Nuclei were stained with DAPI Solution (1 mg/mL) (Thermofisher).

Imaging

Brightfield and fluorescent images were obtained using a Leica DM6000 B Microscope. Confocal microscopy was performed using a Leica SP8X. Images were processed and quantified using ImageJ (NIH).

Quantification and statistical analysis

Unless otherwise specified in the figure legends, all experiments reported in this study were repeated at least three independent times. Statistical analyses were performed in GraphPad Prism 5. Significance and the value of n were calculated with the indicated methods in each figure legend. The data are presented as the mean ± SD. Asterisks in figures indicate the following: * = p-value < 0.05, ** = p-value < 0.005; *** = p-value < 0.0005; **** = p-value < 0.00005. Horizontal lines within each box of all the boxplots represent median values, box heights symbolize the interquartile range (IQR) (IQR = Q3–Q1); Q3, whiskers represent values up to 1.5 times IQR greater than Q3 (top: Q3 + 1.5 × IQR) or smaller than Q1 (bottom: Q1 – 1.5 × IQR).

SUPPLEMENTAL REFERENCES

Hess, J., Unger, K., Orth, M., Schötz, U., Schüttrumpf, L., Zangen, V., Gimenez-Aznar, I., Michna, A., Schneider, L., Stamp, R., et al. (2017). Genomic amplification of Fanconi anemia complementation group A (FancA) in head and neck squamous cell carcinoma (HNSCC): Cellular mechanisms of radioresistance and clinical relevance. Cancer Lett. *386*, 87–99.

Kurmann, A.A., Serra, M., Hawkins, F., Rankin, S.A., Mori, M., Astapova, I., Ullas, S., Lin, S., Bilodeau, M., Rossant, J., et al. (2015). Regeneration of Thyroid Function by Transplantation of Differentiated Pluripotent Stem Cells. Cell Stem Cell *17*, 527–542.
